# Investigating the Influence of Vaccine Literacy, Vaccine Perception and Vaccine Hesitancy on Israeli Parents’ Acceptance of the COVID-19 Vaccine for Their Children: A Cross-Sectional Study

**DOI:** 10.3390/vaccines9121391

**Published:** 2021-11-24

**Authors:** Yulia Gendler, Lani Ofri

**Affiliations:** The Department of Nursing, School of Health Sciences, Ariel University, Ariel 40700, Israel; laniof@ariel.ac.il

**Keywords:** COVID-19 vaccine, children’s vaccination, vaccine literacy, vaccine hesitancy, vaccine perception, vaccine acceptance

## Abstract

Vaccination is currently the most effective strategy for combating COVID-19. COVID-19 vaccines were introduced to the adult population in Israel in early December 2020 and have been available for children aged 12–15 since June 2021. Our study aimed at assessing the influence of vaccine literacy, perception, hesitancy, and behavior on Israeli parents’ intentions to have their children vaccinated. Using an anonymous online questionnaire, we recruited 520 parents; 70.4% of the parents indicated that they would get their children vaccinated. The participants’ COVID-19 vaccination status was the only socio-demographic factor significantly associated with COVID-19 vaccination acceptability (OR = 32.89; 95%CI = [13.11, 82.54]). The most common sources of information regarding the COVID-19 vaccine were health-care providers and the Internet. Parents who intend to vaccinate their children had higher mean levels of vaccine literacy (2.99 ± 0.47 vs. 3.07 ± 0.44 respectively, *p* = 0.06), more positive perception of the vaccine (mean scores of 2.26 ± 0.75 vs. 3.44 ± 0.68 respectively, *p* < 0.001), and lower perceived vaccine hesitancy (7.53 ± 2.37 vs. 4.68 ± 2.71 respectively, *p* < 0.001) than parents who do not intend to do so. Vaccine behavior was measured using the 5C model of psychological antecedents. All 5C components were significantly correlated with parents’ willingness to vaccinate their children. Understanding of parents’ willingness to have their children receive the COVID-19 vaccine and the barriers to and facilitators of the vaccination is crucial, as vaccination of children aged 5–11 has recently been approved by the FDA. Providing the population with reliable information regarding the COVID-19 vaccine is an important measure in the attempt to increase COVID-19 vaccine acceptance.

## 1. Introduction

The COVID-19 pandemic remains a major worldwide concern with health-related, social and economic impacts [1]. To date, approximately 254 million cases and more than 5 million deaths from COVID-19 have been registered around the world, and approximately 1.3 million confirmed cases and more than 8000 deaths in Israel alone [2]. In order to mitigate morbidity and mortality, several safe and effective vaccines against COVID-19 have been developed. Vaccination benefits both the individual who receives it through direct protection, and greater society through community protection [3,4]. The first mass vaccination program of population aged 16 and above in Israel started in early December 2020. The vaccination routine included usage of Pfizer–BioNTech’s mRNA COVID-19 vaccine BNT162b2 (Tozinameran), with a two-dose schedule having a 21-day interval between doses [5]. As of 1 June 2021, 81% of the Israeli population aged ≥16 years had received the second vaccine. Vaccine uptake among the adult population was high, exceeding 75% among those aged 20 years and increasing with age, reaching 90% in individuals aged ≥60 years [6].

Children of all ages are susceptible to COVID-19 infection. Although the majority of cases of COVID-19 in children younger than 16 are asymptomatic or mild, there are reports regarding significant morbidity with a need for hospitalization and intensive care in this population [3,7]. In addition to the direct health-related effect, children carry a substantial social and mental health-related burden [8]. Following FDA approval, provided in June 2021, a vaccination program of children aged 12–15 was implemented in Israel [9]. As of the time of this report, the necessity of policy mandating use of a COVID-19 vaccine among children aged 12–15 in Israel is still controversial [10], and many parents are reluctant to vaccinate their children [4].

Vaccine hesitancy, a behavior of delay in acceptance or refusal to vaccinate despite available services, exists in many countries, including Israel [4,11]. The World Health Organization (WHO) has considered this phenomenon to be a global health threat since 2019 [12]. Previous studies have reported worrying rates of parent hesitancy regarding routing childhood vaccines, in addition to influenza and human papillomavirus vaccines [13,14]. Vaccine hesitancy affects the hesitant individual and, consequently, the entire community, as a high coverage rate is necessary to achieve the herd immunity needed to flatten the epidemic curve [15]. The levels of uncertainty regarding the safety and efficacy of the COVID-19 vaccine are extremely high, and are exacerbated by an excess of contradictory information [16]; therefore, COVID-19 vaccine hesitancy is anticipated [4].

Vaccine hesitancy is multidimensional and specific to one’s environment, setting and vaccine characteristics. Several theoretical models examined the psychological underpinnings of vaccine hesitancy [17] and behavior [11]. The framework of 5C psychological antecedents of vaccination includes: 1. Confidence (trust in vaccine effectiveness, safety and necessity, and the system that delivers it); 2. Complacency (perceived the disease as low risk, vaccination not seen as necessary); 3. Constraints (perceived low vaccine availability, affordability and accessibility); 4. Calculation (individuals’ engagement in information searching); and 5. Collective responsibility (willingness to take the vaccine in order to protect others through herd immunity) [11,18,19].

As in other countries, the need for immunization in Israel is highly covered by the media. In addition to the adjustment and sensitization of the general population, the media can also cause negative effects by creating massive informative overload and/or providing unauthorized medical advice [16,20]. Therefore, the health literacy of the general population is exceptionally important.

Health literacy is defined as a “personal, cognitive and social skill that determines the capability of an individual to access, understand, and use information to improve and maintain personal health” [20,21]. The basic assumption of this definition is that people with a satisfactory level of health literacy manage their health more efficiently [20]. However, contrary to expectations, studies that examined vaccine acceptance have shown that parents with higher skills appeared to be more at risk of not vaccinating their children. Good levels of education did not always correspond to suitable abilities of critical interpretation of the information: people with appropriate levels of functional, interactive or even critical literacy could risk incurring errors of evaluation, sometimes due to an overload of information [22]. Based on these findings, the concept of vaccine literacy (VL) was developed by Biasio et al. [16,23]. They defined vaccine literacy not only as a level of knowledge about vaccination, but also as a system with decreased complexity to communicate and offer vaccines as a necessity in a functioning health system [20,22]. Nevertheless, when reviewing the factors of vaccine hesitancy, limited vaccine literacy is rarely taken into account [22].

Given the prevalence of the COVID-19 disease, and the importance of acceptance of vaccination that will prevent severe disease in children, it is important to examine the factors that influence parents’ acceptance of the COVID-19 vaccine for their children. Previous studies have found that factors such as anxiety regarding COVID-19, parents’ education and influenza vaccination status have influenced parents’ willingness to have their children vaccinated [24,25]. Knowledge about the COVID-19 vaccine was found to be correlated with vaccine acceptance among the undergraduate population in Italy. Galle’ et al. also highlighted the association between sources of information and the level of knowledge regarding the COVID-19 vaccine [26]. These findings emphasize that COVID-19 vaccine acceptance is a multidimensional phenomenon, and needs to be investigated as such. However, a clear connection between vaccine literacy, vaccine perception and vaccine hesitancy, and parents’ decision about vaccination in children, has not yet been established.

Our study objectives were to assess the influence of vaccine literacy, perception, hesitancy and behavior on Israeli parents’ intentions to vaccinate their children aged 12–15 against COVID-19. We also assessed the sources of information the parents utilize to receive information regarding the COVID-19 vaccine for children.

## 2. Methods

### 2.1. Setting

In June 2021, we conducted a rapid cross-sectional study using an anonymous online questionnaire. A URL linking users to the Qualtrics™ questionnaire was distributed via public Facebook pages of parents’ groups, aimed at approximately 50,000 eligible respondents. These groups consist of parents to school-aged children from all over the country, representing all national and religious affiliations, potentially reducing possible selection bias. Respondents were asked to provide honest answers, were not given any incentives for participation, and could reply only once to the survey.

### 2.2. Participants

The survey was aimed at Hebrew-speaking adult individuals who were parents to children aged 12–15 years. No other exclusion criteria were applied.

### 2.3. Ethical Considerations

Approval was obtained from the Institutional Review Board of Ariel University (approval code: AU-HEA-YG-20210611). Written informed consent was received online before the respondents began responding to the questionnaire.

### 2.4. Measures

The questionnaire was composed of 47 questions, including:

- Demographic data: age, gender, place of birth, area of residency, education, occupation, religious affiliation.
- Participants’ vaccination status—we asked the participants to indicate whether they had been vaccinated for COVID-19.
- Participants were asked to indicate which sources of information they had used while searching for information regarding the COVID-19 vaccine. Response options included: family, friends, healthcare professionals, television, internet, newspapers, medical journals, and social media, or “didn’t look for information” (multiple answers were accepted).
- Vaccine literacy was measured using a VL scale [23,27], composed of 12 Likert-type questions. Four items were aimed at assessing functional VL and eight items evaluated interactive-critical VL. Each response was rated with a 4-point Likert scale (4—never, 3—rarely, 2—sometimes, 1—often, for the functional questions; and 1—never, 2—rarely, 3—sometimes, 4—often, for the interactive-critical questions). The score was obtained from the mean value of the answers to each scale (a range of 1–4), a higher value corresponding to a higher VL level.
- COVID-19 vaccine perception was measured using seven Likert-type questions. Each response was rated with a 4-point Likert scale (1—strongly disagree, 4—strongly agree). Examples of items: “There is no need to vaccinate children against COVID-19, as herd immunity has already been achieved in Israel”; “There is no need to vaccinate children against the corona virus, as the disease in children is usually mild”. Mean score of items under each domain were computed, a higher value corresponding to a negative perception of the COVID-19 vaccine.
- Vaccination behavior was measured using a 15-item tool developed from a “5C model” of psychological antecedents to vaccination [19]. Each of the five antecedents, including confidence, complacency, constraints, calculation and collective responsibility, was assessed by three rating items on a 5-point scale (5—strongly agree, 1—strongly disagree). A mean score of items under each domain was computed, with a higher average score indicating stronger agreement regarding the corresponding domain.
- Perceived vaccine hesitancy was measured by a single item asking the participants to grade to what extent they are hesitating to vaccinate their children on an 11-point Likert scale (0 = not hesitant at all, 10 = very hesitant).
- COVID-19 vaccination intention was measured by two items: first we asked the participants if they would be willing to vaccinate their children against COVID-19 (very likely/somewhat likely/somewhat unlikely/definitely not), and then how likely they would be to vaccinate their children on an 11-point Likert scale (0 = definitely no, 10 = definitely yes). When a dichotomous division was required, we coded “very likely/somewhat likely” as “intent to vaccinate” and “somewhat unlikely/definitely not” as “does not intent to vaccinate”.

After receiving approval from the authors of the original questionnaires, the instruments measuring COVID-19 vaccine literacy and the 5C model of vaccine antecedents were translated to Hebrew by two independent experts. Back-translation of the instruments to English was performed by another expert, who did not participate in the original translations. A further validation process was implemented: experts who were familiar with the construct that the questionnaire was designed to measure were asked to judge, as a panel, whether the questionnaire items adequately measured the construct intended to be assessed, and whether the items were sufficient to measure the domain of interest.

### 2.5. Data Analysis

To estimate the required sample size, we conducted an a priori power analysis. Based on the population of children aged 12–15 years in Israel (N = ~800,000), with 95% confidence levels, and 4% margin of error, a total of 496 participants were needed for the study. Data were extracted from the Qualtrics™ software and analyzed using IBM SPSS version 26. Qualitative data are presented as frequencies and percentages; quantitative variables are presented as mean and standard deviation, and an independent samples t-test was used for analysis. Correlation between the variables were analyzed using a Pearson test. Binary logistic regression was applied to predict factors affecting COVID-19 vaccine acceptability among parents. The effect of these factors on parents’ intention to vaccinate their children was analyzed using a linear regression. The internal consistencies of the VL, 5C and vaccine perception questionnaires were assessed through Cronbach’s alpha coefficient (see Supplementary materials S1). Effect size was measured using Cohen’s *d* for two independent samples. Statistical significance was set at *p* < 0.05.

## 3. Results

Table 1 presents overall participant characteristics (*n* = 520) and their bivariate association with COVID-19 vaccine acceptance among parents. The mean age of the sample was 44.76 ± 8.09 years; 401 (77.1%) were female; 208 (40%) defined themselves as secular, 76 (14.6%) as traditional, 170 (32.7%) as religious, and 66 (12.7%) as Jewish Orthodox. Most participants held an academic degree (73.7%), and 34.4% were healthcare workers. COVID-19 vaccination uptake rate among responding parents was 76%. COVID-19 vaccination status of the participants was the only univariate determinant associated with COVID-19 vaccination acceptability for their children (OR = 32.89; 95%CI = [13.11, 82.54]). Among parents who had already received the COVID-19 vaccine (Table 2), 61% indicated their intent to get the vaccine for their children; conversely, among parents who stated that they had not been vaccinated, only 9.4% responded that they intend to get their children vaccinated.

**Figure 1:**
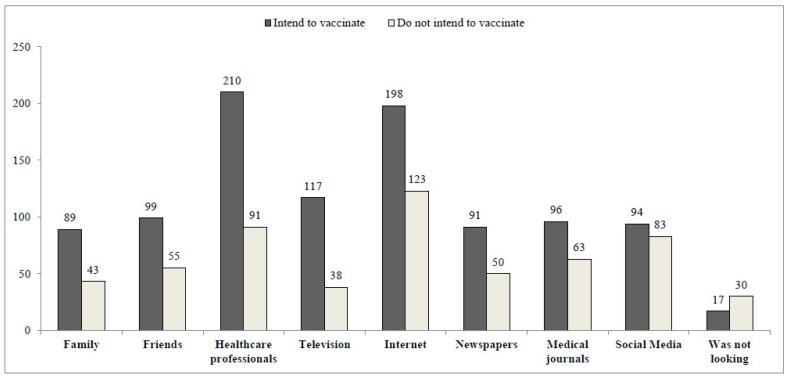
COVID-19 vaccine information sources, according to vaccination acceptance. Crude numbers, not mutually exclusive.

Figure 1 presents the difference in sources of information regarding the COVID-19 vaccine utilized by respondents who aim to vaccinate their children as compared to respondents who do not intend to do so. The information source most frequently used by the respondents who intend to vaccinate their children was healthcare professionals (210 responses), whereas participants who do not intend to vaccinate their children mostly retrieve information from the Internet (123 responses).

Of 520 participants, 366 (70.4%) indicated that they are “very likely” or “somewhat likely” to have their children receive the COVID-19 vaccine (Table 3). Parents who indicated that they are likely to vaccinate their children had higher mean scores of functional, interactive/critical skills and total VL scores (non-significant differences). COVID-19 vaccine perception mean score was higher among parents who did not intend to vaccinate their children, indicating a more negative perception of the vaccine compared to parents who intend to vaccinate their children (3.44 ± 0.68 vs. 2.26 ± 0.75 respectively, *p* < 0.001). Parents who intend to get their children vaccinated had higher scores of the “confidence”, “calculation” and “collective responsibility” components, and lower scores of the “complacency” and “constraints” components of the 5C model.

**Figure 2:**
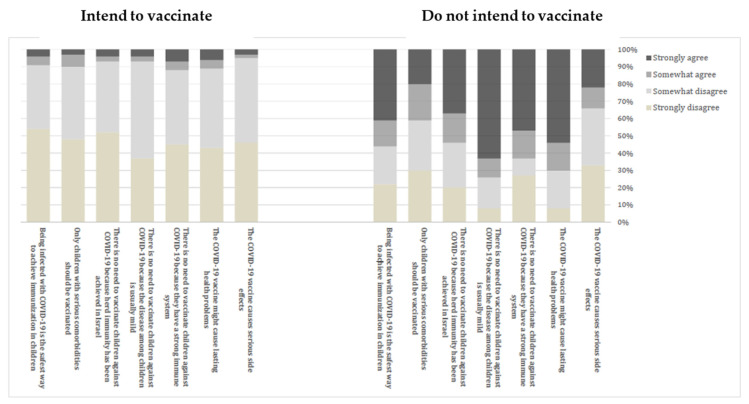
Parents’ perception of COVID-19 vaccine—difference between parents who intend to vaccinate their children and parents who do not intend to vaccinate.

Figure 2 presents parents’ perceptions of the COVID-19 vaccine for children, stratified by whether they do or do not intend to get their children vaccinated. Parents who indicated that they would not vaccinate their children were far more likely to agree that the COVID-19 vaccine might cause serious side effects and lasting health problems, and that there is no need to vaccinate children against COVID-19 because they have a strong immune system, the disease among children is usually mild, and herd immunity has already been achieved in Israel. A large proportion of those parents believed that only children with serious comorbidities should be vaccinated, and that being infected with COVID-19 is the safest way to achieve immunization in children.

**Figure 3:**
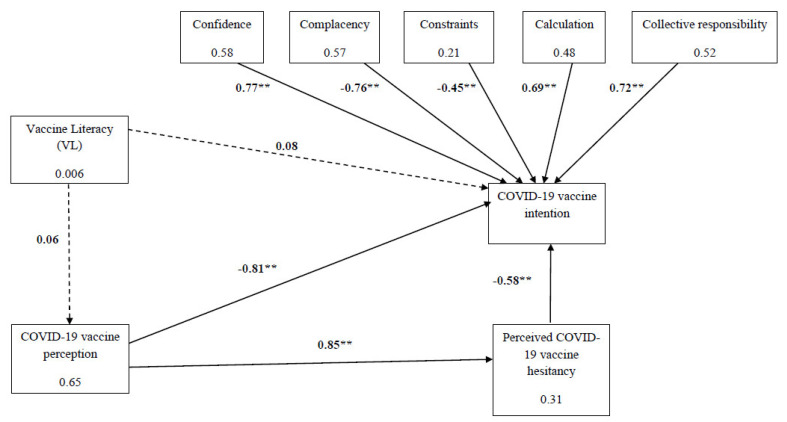
The effect of 5C, vaccine literacy, perceived vaccine hesitancy and COVID-19 vaccine perception on COVID-19 vaccination intention. Values on the errors are correlation coefficients (β) and values in the boxes are r^2^; ** *p* < 0.001.

Figure 3 presents the direct effect of VL, COVID-19 vaccine perception, and the 5C components of vaccine behavior and perceived COVID-19 vaccine hesitancy on parents’ intention to vaccinate their children. Participants’ VL was not correlated with COVID-19 vaccine perception or with COVID-19 vaccine intention. Vaccine perception had a high and significant correlation with COVID-19 vaccine intention (r = −0.58, *p* < 0.001). Lower scores of vaccine perception (indicating a more positive approach) were strongly correlated with higher intention to vaccinate. COVID-19 vaccine perception was correlated with perceived COVID-19 vaccine hesitancy (r = 0.85, *p* < 0.001). Higher scores of vaccine perception (indicating a more negative approach) were correlated with high perceived vaccine hesitancy. High scores of perceived vaccine hesitancy were correlated with lower scores of vaccination intention (r = −0.78, *p* < 0.001). All five components of the 5C model of vaccine behavior were significantly correlated with COVID-19 vaccine intention.

## 4. Discussion

Our cross-sectional study was conducted at the time the COVID-19 vaccine was approved for children aged 12–15 years in Israel and was one of the first studies to investigate the factors that influence parents’ willingness to vaccinate the children. Seventy-one percent of parents in our sample were willing to allow their children to receive the COVID-19 vaccine. Numerous studies have presented a wide range of parental acceptability rates of the COVID-19 vaccines for children, ranging from around 40% in Turkey, 60% in the U.S., and above 80% in New Zealand and England [28,29]. In order to further improve the acceptability rate among Israeli parents, it is important to understand the reasons for parents’ willingness or reluctance to have the COVID-19 vaccine administrated to their children.

Previous studies have shown that parents’ intentions to vaccinate their children against COVID-19 were associated with child and caregiver demographics, such as age, gender, marital status, years of education and income, in addition to flu vaccination history [24,28]. Other studies have reported that educational level did not directly affect parents’ intention to vaccinate their children [25]. Healthcare workers were more likely to agree to vaccinate themselves and their children than non-healthcare worker parents [30]. In the present study, all of these factors were also taken into account. A logistic regression model demonstrated that the only factor that was significantly associated with parents’ acceptability of COVID-19 vaccine for their children was their own COVID vaccination status. Parents who had already received the COVID-19 vaccine were more likely to have their children vaccinated than parents who did not receive the vaccine.

In Israel, as in many other countries, information and updates regarding the COVID-19 outbreak and vaccines development are widely covered by the media. Health communication is the most effective way to convey information and encourage behavior modifications in order to prevent morbidity and mortality [31]. Health communication strategies utilizing mass media and social networks have been associated with health beliefs and behaviors [32]. However, previous studies have demonstrated that most individuals continue to rely more on medical staff as a source for correct and updated health information [33]. Our study compared the sources of information utilized by parents who intend to vaccinate their children against COVID-19 compared to parents who do not intend to do so. Parents who reported that they intend to vaccinate their children were more likely to rely on the medical staff as a trusted source of information, whereas parents who did not intend to do so often relied on the Internet. These findings were consistent with results of an American national survey, which demonstrated that health care providers were the most trusted source of information regarding the COVID-19 vaccine [13]. Today, the Internet is a widely used source of information, due to its high availability and accessibility. However, findings from the Health Information National Trend Survey (HINTS) in the US demonstrated that individuals who distrusted their health care provider were more likely to use the Web, but less likely to perceive the information gleaned from searches as useful [32].

Information provided by mass media often results in controversies and is sometimes abundant with fake news [16]. Therefore, health literacy—the population’s ability to collect and understand information—is extremely important. Previous studies have demonstrated a paradox, discovering that individuals with high levels of health literacy were less likely to fully comply with child vaccination programs. This phenomenon can be explained by the overexposure of a population with a high health literacy to misleading information, especially via the Internet and social media [16,34]. The rapid emergence of COVID-19 and the swift development of vaccines against it have raised the need to redefine the concept of health literacy. The newly developed concept of vaccine literacy (VL) was constructed on the same basis as health literacy, and was validated and tested in previous studies [16,23]. After examining it in our study in the context of parental acceptability COVID-19 vaccination for their children, we found no association between VL and parents’ willingness to vaccinate their children.

Previous studies suggested that VL is associated with vaccine perception. The amount and variety of information conveyed in the media and on the Internet was characterized by controversies and fake news [16,35]. In our findings, parents who reported a positive perception of the vaccine were less likely to have their children vaccinated. Parents who were not willing to vaccinate their children indicated that they perceive the COVID-19 vaccine as less safe. Those parents were particularly concerned with the immediate and long-term side effects of the vaccine, and they believed that having the children vaccinated is unnecessary because children have stronger immune systems and the disease among children is usually mild. A large proportion of them believed that herd immunity had already been achieved in Israel, making the vaccine superfluous.

Vaccine hesitancy has been identified by the WHO as one of the top ten threats to global health in 2019 [36]. Previous pandemics of influenza had raised the awareness of the vaccine hesitancy concept, because extremely low acceptance rates to newly introduced flu vaccines had been noted. Hence, there was a justified concern that hesitancy regarding the COVID-19 vaccine will hinder global attempts to control the current pandemic, resulting in major adverse health and socioeconomic consequences [15]. Vaccine hesitancy is a multidimensional phenomenon associated with one’s attitudes and beliefs, in addition to environmental, social, cultural and political factors [37,38]. Our study examined participants’ report of their perceived vaccine hesitancy, and the psychological antecedents of vaccination. Parents who reported high levels of perceived vaccine hesitancy were less inclined to get their children vaccinated. The 5C framework described vaccine hesitancy as prompted by five major factors: confidence, complacency, constraints, calculation and collective responsibility. Our results demonstrated that parents with high scores of confidence, calculation and collective responsibility (i.e., they trust vaccine effectiveness and safety, were engaged in information searching and were willing to protect others) were more likely to agree to vaccinate their children. Parents with high scores of complacency and constraints (i.e., they perceived the disease as low risk and the vaccine as non-accessible and even unnecessary) were less likely to get their children vaccinated.

Our research highlighted many factors that may influence parents’ willingness to vaccinate their children against COVID-19. These factors include their perceived hesitancy dictated by their beliefs and values, their personal experience with the COVID-19 vaccine, their perception of the vaccine and the media from which they draw information. Previous studies have demonstrated that knowledge regarding the COVID-19 vaccine is significantly related to vaccine acceptance. Providing the population with accurate knowledge may reduce vaccine hesitation, thus increasing compliance to COVID-19 vaccination [26]. In a world of information overload, it is sometimes difficult to separate reliable information from fake news. Thus, public health information campaigns should be broadcast in all the media, including the Internet, and should aim at raising awareness of vaccination against COVID-19. Many parents in our sample reported that they draw information regarding the COVID-19 vaccine from healthcare providers. Therefore, efforts should be made to provide medical staff with up-to-date information regarding the COVID-19 vaccine for children. Appropriate medical recommendations and encouragement from the physician may enhance a more positive perception of the vaccine and reduce parents’ hesitancy.

## 5. Limitations

This study had some limitations: The self-report nature of online surveys may have affected the responsiveness rates, potentially causing selection bias. The URL link was distributed via Facebook, naturally aiming for the more educated population (73.7% of the participants held an academic degree, 34.4% were healthcare workers; 76% were vaccinated). Therefore, it is unclear to what extent our findings are generalizable to the entire Israeli population. Due to the cross-sectional study design, the temporal ordering of the variables could not be established; thus, causation between variables cannot be fully indicated. Moreover, there are many other participant characteristics that could have played a role regarding their acceptance of vaccination for their children (e.g., pre-existing vaccine hesitancy, adverse reaction from vaccines, family member who had been infected with or had even died from COVID-19). Despite these limitations, our study is among the first studies exploring the factors associated with parents’ acceptance of children vaccination against COVID-19.

## 6. Conclusions

As the children’s legal guardians, parents have the right to decide whether their children should be vaccinated. However, in the midst of a global pandemic, a different approach to the decision-making process of the parents regarding children vaccination against COVID-19 is required. Understanding of parents’ willingness to have their children receive the COVID-19 vaccine, and the barriers to and facilitators of COVID-19 vaccination, is crucial. All of these are especially important because vaccines for children ages 5–11 have been approved by the FDA. National efforts should be directed to providing reliable information regarding the COVID-19 vaccine, including its immediate and long-term side effects. These means may improve the general population’s vaccine perception. In addition, efforts to combat vaccine hesitancy are essential, both for eradicating the current pandemic and for increasing responsiveness to childhood vaccines in general.

## Figures and Tables

**Table 1:** Socio-demographic characteristics and crude odds ratios predicting COVID-19 vaccine acceptance among parents to children aged 12–15 (*N* = 520).

Predictor			COVID-19 Vaccination Acceptance	
		Number (%)	OR [95%CI]	*p* Value
Gender	Female	401 (77.1)	1.67 [0.92, 3.03]	0.09
	Male	119 (22.9)	[1]	
Age, estimate for 1 year	Mean (±SD)	44.76 (8.09)	1.02 [0.98, 1.06]	0.15
Place of birth	Israel	377 (72.5)	1.19 [0.60, 2.36]	0.94
	Other	143 (27.5)	[1]	
Area of residency	Central Israel	251 (48.3)	1.13 [0.65, 1.96]	0.66
	Other	269 (51.7)	[1]	
Education	Academic	383 (73.7)	1.41 [0.63, 3.05]	0.41
	Other	137 (26.3)	[1]	
Occupation	Healthcare workers	179 (34.4)	1.36 [0.87, 2.13]	0.18
	Other	341 (65.6)	[1]	
Religious affiliation	Secular	208 (40.0)	1.05 [0.59, 1.86]	0.87
	Other	312 (60.0)	[1]	
COVID-19 vaccination status	Yes	395 (76.0)	32.89 [13.11, 82.54]	<0.001
	No	125 (24.0)	[1]	

COVID-19 vaccination acceptance among parents is considered a positive response to the question “Will you be willing to vaccinate your child against COVID-19?”

**Table 2:** Parents’ intention to vaccinate their children based on their own COVID-19 vaccination status.

	Parents’ Intention to Vaccinate Their Children	
	Yes	No
Parents’ vaccination status (%)		
Yes	317 (61.0%)	78 (15.0%)
No	49 (9.4%)	76 (14.6%)

**Table 3:** Mean VL functional, interactive and total scores, COVID-19 vaccine perception, the 5C components of vaccine behavior, and perceived vaccine hesitancy, according to COVID-19 vaccination acceptance.

Variable Mean Scores (±SD)	Likelihood to Vaccinate Their Children against COVID-19		*p* Value	Cohen’s *d*
	Very Likely/Somewhat Likely (*n* = 366)	Very Unlikely/Definitely not (*n* = 154)		
VL Functional skills	3.27 (±0.61)	3.18 (±0.60)	0.13	0.15
VL Interactive/critical skills	2.86 (±0.66)	2.80 (±0.62)	0.31	0.09
VL Total	3.07 (±0.44)	2.99 (±0.47)	0.06	0.18
COVID-19 vaccine perception	2.26 (±0.75)	3.44 (±0.68)	<0.001	1.68
5C Model:				
Confidence	3.81 (±1.13)	2.06 (±1.10)	<0.001	1.60
Complacency	2.17 (±1.08)	3.86 (±1.09)	<0.001	1.56
Constraints	2.55 (±0.98)	3.33 (±0.94)	<0.001	0.81
Calculation	3.19 (±0.95)	1.88 (±0.84)	<0.001	−1.43
Collective responsibility	3.96 (±1.04)	2.51 (±1.06)	<0.001	−1.39
Perceived COVID-19 vaccine hesitancy	4.68 (±2.71)	7.53 (±2.37)	<0.001	1.09

## Data Availability

The data at the basis of the findings of this study are available on request from the corresponding author.

## Supplementary Materials

The following are available online at www.mdpi.com/article/10.3390/vaccines9121391/s1, Supplementary File S1: Tools employed to assess vaccine literacy, COVID-19 vaccine perception and psychological antecedents to vaccination
