# Supplementary material for: Investigating the Influence of Vaccine Literacy, Vaccine Perception and Vaccine Hesitancy on Israeli Parents’ Acceptance of the COVID-19 Vaccine for Their Children: A Cross-Sectional Study
Source: Vaccines (Basel). 2021 Nov 24;9(12):1391. doi: 10.3390/vaccines9121391 (PMC8703688; doi:10.3390/vaccines9121391)
Supplement: Supplementary file 1 [file vaccines-09-01391-s001.zip › Supplementary materials.pdf]

Tools employed to assess vaccine literacy, COVID-19 vaccine perception and psychological antecedents to vaccination

Variable (Cronbach's alpha)	Measure and items	Assessment (score)
VL functional skills (α=0.82)	When reading or listening to information about COVID-19 vaccines:<br>1. Did you find words you didn't know?<br>2. Did you find that the texts were difficult to understand?<br>3. Did you need much time to understand them?<br>4. Did you need someone to help you understand them?	Ordinal, 4 points Likert scale for frequency:<br>Often (1), Sometimes (2), Rarely (3), Never (4)
VL interactive / critical skills (α=0.77)	When looking for information about COVID-19 vaccines:<br>1. Have you consulted more than one source of information?<br>2. Did you find the information you were looking for?<br>3. Have you had the opportunity to use the information?<br>4. Did you discuss what you understood about COVID-19 vaccination in children with a healthcare provider?<br>5. Did you consider whether the information collected was about your child?<br>6. Have you considered the credibility of the sources?<br>7. Did you check whether the information was correct?<br>8. Did you find any useful information to decide on whether or not to get your child vaccinated?	Ordinal, 4 points Likert scale for frequency:<br>Often (4), Sometimes (3), Rarely (2), Never (1)
COVID-19 vaccine perception (α=0.80)	How much do you agree with the following statements?<br>1. The COVID-19 vaccine causes serious side effects<br>2. The COVID-19 vaccine might cause lasting health problems<br>3. There is no need to vaccinate children against COVID-19 because they have a strong immune system<br>4. There is no need to vaccinate children against COVID-19 because the disease among children is usually mild<br>5. There is no need to vaccinate children against COVID-19 because herd immunity has been achieved in Israel	Ordinal, 4 points Likert scale for agreement:<br>Strongly agree (4), Agree (3), Disagree (2), Strongly disagree (1)

	6. Only children with serious comorbidities should be vaccinated<br>7. Being infected with COVID-19 is the safest way to achieve immunization in children	
The 5C psychological antecedents of vaccination	How much do you agree with the following statements?	Ordinal, 5 points Likert scale for agreement:<br>Strongly agree (5), Agree (2), Neutral (3), Disagree (2), Strongly disagree (1)
Confidence (α=0.86)	1. I am completely confident that COVID-19 vaccines are safe<br>2. I am completely confident that COVID-19 vaccines are effective<br>3. Regarding COVID-19 vaccines, I am confident that public authorities decide in the best interest of the community	
Complacency (α=0.85)	4. Vaccination against COVID-19 for children is unnecessary<br>5. The immune system of children is so strong; it also protects them against COVID-19<br>6. COVID-19 disease in children is not severe; vaccinate them is superfluous	
Constrains (α=0.78)	7. Everyday stress prevents me from having my child vaccinated against COVID-19<br>8. For me, it is inconvenient to have my child vaccinated against COVID-19<br>9. Visiting the doctor makes me feel uncomfortable; this keeps me from having my child vaccinated against COVID-19	
Calculation (α=0.75)	10. When I think about having my child vaccinated against COVID-19, I weigh its benefits and risks to make the best decision possible<br>11. I closely consider whether COVID-19 vaccine is useful for my child	

	12. It is important for me to fully understand the topic of vaccination before I get my child vaccinated
Collective responsibility (α=0.85)	13. Like everyone else, I must get my child vaccinated<br>14. Having my child vaccinated against COVID-19 can protect people with a weaker immune system<br>15. Vaccination against COVID-19 is a collective action to prevent the spread of the disease
